# Vitamin D status and adiposity in children with low-energy fractures: a prospective case–control study

**DOI:** 10.1007/s00431-026-07509-x

**Published:** 2026-10-09

**Authors:** Andrea Cosentino, Daniel Weghuber, Pier Francesco Indelli, Nicole Fantini, Wilhelm Berger, Olaf Stefan Schmidt, Wolfgang Högler

**Affiliations:** 1 Paracelsus Medical University, Strubergasse 21, 5020 Salzburg, Austria; 2 Dolomiti Sportclinic, 39046 St. Ulrich (BZ), Italy; 3 Department of Orthopedics and Traumatology, Hospital of Merano (SABES-ASDAA), Via Rossini 5, 39012 Merano-Meran, Italy; 4 Department of Pediatrics, Paracelsus Medical University, Strubergasse 21, 5020 Salzburg, Austria; 5 Institute of Biomechanics, Paracelsus Medical University, Strubergasse 21, 5020 Salzburg, Austria; 6 The Breyer Center for Overseas Studies in Florence, Stanford University, 50125 Florence, Italy; 7 Department of Pediatrics, Hospital of Merano (SABES-ASDAA), Via Rossini 5, 39012 Merano-Meran, Italy; 8 Centre for Growth and Osteology, Department of Paediatrics and Adolescent Medicine, Johannes Kepler University, 4020 Linz, Austria

**Keywords:** Pediatric fracture, Adiposity, Body mass index, Vitamin D, Bone mineralization, Parathyroid hormone, Alkaline phosphatase, Low-energy trauma, Calcium metabolism, Pediatric bone health

## Abstract

Fractures in children are influenced by both skeletal biology and mechanical factors. While alterations in vitamin D and other metabolic markers have been proposed as contributors to fracture risk, their role in predicting fractures in otherwise healthy pediatric populations remains unclear. This study aimed to determine whether biochemical markers of bone metabolism and body mass are independently associated with fracture occurrence and fracture patterns in children after low-energy trauma and to assess their impact on fracture patterns and anatomical distribution. We conducted a prospective case–control study including 100 children presenting after low-energy trauma (50 with radiographically confirmed fractures and 50 controls). Serum 25OH vitamin D (25OHD), parathyroid hormone (PTH), calcium, phosphate, and alkaline phosphatase (ALP) were measured within 1 week of presentation. Associations with fracture occurrence were assessed using multivariable logistic regression, including age, sex, body mass index z-score (BMI-z), an interaction term between 25OHD and BMI-z, and an additional exploratory model evaluating a 25OHD × season interaction. The discriminatory ability of 25OHD was evaluated using receiver operating characteristic analysis. Children with fractures had lower serum 25OHD concentrations and a higher prevalence of levels below 50 nmol/L compared with controls. Other biochemical markers were largely within normal ranges, although ALP levels were modestly higher in fracture patients. In multivariable analysis, 25OHD levels below 50 nmol/L were associated with increased odds of fracture, whereas age and BMI-z were not independently associated with fracture occurrence. However, a significant interaction between 25OHD and BMI-z was observed, indicating that the association between vitamin D status and fracture risk is influenced by adiposity. The overall discriminatory ability of 25OHD for fracture risk was modest.

*Conclusion*: Lower 25OHD levels were observed in children with low-energy fractures. The association between vitamin D status and fracture risk appears to be modest and influenced by adiposity, with a more pronounced effect at higher BMI-z levels. Therefore, fracture susceptibility in otherwise healthy children is likely driven primarily by biomechanical and developmental factors rather than vitamin D status, supporting a targeted rather than routine laboratory assessment of metabolic markers in children presenting with fractures.

*ClinicalTrials.gov Identifier*: NCT07143552.

**Table:** 

**What is Known:** • *Lower serum 25-hydroxyvitamin D concentrations have been reported in children with fractures, but the relationship between vitamin D status and fracture risk remains controversial.* • *Childhood obesity is associated with lower circulating vitamin D concentrations and may independently influence fracture susceptibility.*	
**What is New:** • *Vitamin D insufficiency (< 50 nmol/L) was associated with increased odds of low-energy fractures in children, although its overall discriminatory ability was modest.* • *The association between vitamin D status and fracture risk was modified by adiposity, with a stronger relationship observed in children with higher BMI-z.* • *Biochemical markers of bone mineralization were largely normal, supporting the concept that fracture susceptibility in otherwise healthy children is driven predominantly by biomechanical and developmental factors rather than clinically relevant metabolic bone disease.*

**What is Known:**

• *Lower serum 25-hydroxyvitamin D concentrations have been reported in children with fractures, but the relationship between vitamin D status and fracture risk remains controversial.*

• *Childhood obesity is associated with lower circulating vitamin D concentrations and may independently influence fracture susceptibility.*

**What is New:**

• *Vitamin D insufficiency (< 50 nmol/L) was associated with increased odds of low-energy fractures in children, although its overall discriminatory ability was modest.*

• *The association between vitamin D status and fracture risk was modified by adiposity, with a stronger relationship observed in children with higher BMI-z.*

• *Biochemical markers of bone mineralization were largely normal, supporting the concept that fracture susceptibility in otherwise healthy children is driven predominantly by biomechanical and developmental factors rather than clinically relevant metabolic bone disease.*

## Introduction

Fractures during childhood are common and represent an important clinical and public health issue. Epidemiological data suggest that approximately half of boys and up to 40% of girls sustain at least one fracture before reaching adulthood [1]. The mechanical competence of the growing skeleton is influenced by multiple factors, including bone mineral content, structural organization of bone tissue, cellular activity, muscle forces, and the mechanical loads applied during daily activities. Evidence from the Avon Longitudinal Study of Parents and Children (ALSPAC) has demonstrated that reduced bone mass in childhood is associated with an increased likelihood of fractures, indicating that skeletal fragility can already be detected during early life [2].

Alongside intrinsic skeletal characteristics, broader physiological and lifestyle factors may also contribute to fracture susceptibility in children. In particular, the worldwide increase in childhood overweight and obesity has raised concerns regarding its potential impact on musculoskeletal health [3]. Since the 1970 s, the prevalence of childhood obesity has risen dramatically and is now recognized as a major global health challenge, with consequences extending to metabolic, cardiovascular, and psychosocial domains [4–6]. Excess adiposity is also associated with lower circulating levels of 25-hydroxyvitamin D (25OHD), partly due to volumetric dilution, storage of vitamin D within adipose tissue, and alterations in metabolic processing [7].

Several large observational studies have reported that children with obesity have a greater risk of fractures and may experience more severe injuries, particularly affecting the lower limbs [8]. Increased body mass can modify fall dynamics and amplify the mechanical forces transmitted to the skeleton during trauma [8, 9]. Although heavier children often have greater absolute bone mass and larger skeletal dimensions, bone strength may not increase proportionally with body weight, potentially leaving the skeleton relatively vulnerable to injury [7, 10, 11].

Reduced circulating 25OHD concentrations are frequently reported in overweight and obese pediatric populations, which may give rise to a perceived relationship between vitamin D status and fracture risk [12, 13]. However, existing evidence remains inconsistent, and several systematic reviews have highlighted the potential role of confounding factors such as adiposity, seasonal variation, and reduced sunlight exposure in explaining this association [14].

Vitamin D is nevertheless essential for maintaining mineral homeostasis and supporting skeletal mineralisation through its effects on intestinal calcium absorption. Marked vitamin D deficiency can lead to rickets or osteomalacia, whereas milder insufficiency in combination with inadequate calcium intake may trigger secondary hyperparathyroidism, increased bone turnover and occult (histological) osteomalacia. Therefore, when assessing skeletal fragility in children, it is important to differentiate between hypomineralization disorders—typically accompanied by elevated parathyroid hormone (PTH) and alkaline phosphatase (ALP)—and quantitative deficits in bone mass [15]. In otherwise healthy children without biochemical abnormalities or insufficient calcium intake, moderate reductions in serum 25OHD are unlikely to substantially compromise bone strength [16, 17]. International consensus guidelines generally consider serum 25OHD concentrations above 50 nmol/L (20ng/ml) adequate to prevent skeletal complications [18], although vitamin D may exert additional physiological effects beyond the skeleton [19, 20].

This study aimed to determine whether biochemical markers of bone metabolism are independently associated with fracture occurrence and fracture pattern, while exploring the interaction between vitamin D status and adiposity.

## Methods

This prospective case–control study investigated metabolic factors associated with pediatric low-energy fractures. Children aged 3–15 years were recruited consecutively between July 2024 and November 2025 at the Orthopedic Department of the Hospitals of Merano and Bressanone. Those presenting after low-energy trauma were invited to participate at the time of their emergency department visit. Fracture cases consisted of children with radiographically confirmed fractures, whereas controls were children presenting after low-energy trauma during the same study period in whom radiographic evaluation excluded a fracture.

Recruitment was performed consecutively during the study period until the target sample size of 100 participants (50 fracture cases and 50 controls) was reached.

Children presenting after high-energy trauma were not considered for enrolment and were therefore not included in the screening process.

Demographic and anthropometric data, including age, sex, height, and weight, were recorded for all participants included in the final analytic cohort. The dependent variable was coded as fracture (1) versus no fracture (0).

Only otherwise healthy children were included. “Otherwise healthy” was defined as the absence of chronic diseases or medications known to affect bone metabolism.

Exclusion criteria comprised:

- High-energy trauma
- Previous diagnosis of metabolic bone disease (including rickets or osteogenesis imperfecta)
- Chronic kidney or liver disease
- Endocrine disorders known to affect bone metabolism (including diabetes mellitus, hyperparathyroidism, and thyroid disease)
- Neuromuscular disorders
- Malignancy
- Chronic inflammatory diseases
- Treatment with medications known to influence bone metabolism (including systemic glucocorticoids and bisphosphonates)
- Inability to obtain informed consent

Information regarding vitamin D supplementation was obtained from parents at enrolment. Vitamin D supplementation was not an exclusion criterion because all reported supplementation was occasional prophylactic use during the winter months, consistent with routine pediatric practice, rather than therapeutic supplementation for an underlying disorder.

Fractures were categorized according to anatomical location to explore potential associations between metabolic markers and fracture patterns. Fracture locations were grouped into wrist/forearm, humerus or supracondylar fractures, femur, tibia, and other fracture sites. For additional analyses, fractures were also classified according to region as upper extremity or lower extremity fractures.

Blood samples were obtained within the first week after injury. The following biochemical markers related to bone metabolism were analyzed: 25OHD, PTH, calcium, phosphate, and ALP. Serum 25OHD concentrations were categorized into vitamin D deficiency < 30 nmol/L, insufficiency 30 to < 50 nmol/L, and sufficiency ≥ 50 nmol/L, according to commonly used clinical thresholds.

Height and weight were obtained from the child’s most recent routine pediatric or sports medicine examination, as reported by the parents in the study questionnaire. Measurements had been performed without shoes according to standard clinical practice. Body mass index (BMI) was calculated as weight (kg)/height (m^2^), and age- and sex-specific BMI z-scores (BMI-z) were calculated using the Centres for Disease Control and Prevention (CDC) BMI Percentile Calculator for Child and Teen. Race/ethnicity was not collected, and height- and weight-for-age z-scores were not calculated.

Venous blood samples were collected within 7 days after presentation and analyzed in the certified laboratories of the Azienda Sanitaria dell’Alto Adige (South Tyrol Health Service). Serum 25-hydroxyvitamin D (25OHD), calcium, phosphate, and alkaline phosphatase (ALP) were measured using Roche Cobas analytical platforms, whereas parathyroid hormone (PTH) was determined using the Roche Cobas Pro immunoassay analyzer. All analyses were performed according to the manufacturers’ instructions and the laboratories’ standard operating procedures, including routine internal and external quality assurance procedures.

To explore the combined effect of adiposity and vitamin D status, an interaction term between BMI-z and vitamin D category was generated. In addition, BMI-z was categorized into tertiles to allow stratified analyses.

Dietary calcium intake was assessed using a food frequency questionnaire adapted from the validated questionnaire developed by Montomoli et al. [21]. The questionnaire was completed by the parents during the Emergency Department visit or, when necessary, at home and returned thereafter. Daily calcium intake (mg/day) was estimated according to the scoring system described by Montomoli et al. [21]. Dietary calcium intake was categorized as adequate or inadequate according to age-specific recommended dietary allowances (700 mg/day for children aged 1–3 years, 1000 mg/day for 4–8 years, and 1300 mg/day for 9–18 years). For interaction analyses, calcium intake was further dichotomized into low and high intake groups based on the sample median.

Age was analyzed both as a continuous variable and grouped into three developmental stages (3–6.99 years, 7–10.99 years, and 11–15.99 years) to explore potential age-related patterns in fracture distribution and metabolic markers.

Statistical analyses were performed using Jamovi (version 2.6) [22–24]. Continuous variables were summarized using means (standard deviation) or median (range). Normality was assessed using the Shapiro–Wilk test. Given the non-normal distribution of several variables, nonparametric tests were applied for most comparisons. Differences between cases and controls were evaluated using the Mann–Whitney *U* test or independent samples *t*-test, as appropriate. Categorical variables were compared using chi-square tests.

Binomial logistic regression analysis was performed to assess the independent association between metabolic markers and fracture occurrence. Predictor variables included age, sex, BMI-z, vitamin D status, and the interaction term between BMI-z and 25OHD status. In addition, serum 25OHD was analyzed as a continuous variable, and an interaction term between 25OHD and BMI-z was included to assess effect modification. Serum 25OHD concentrations were then dichotomized at 50 nmol/L (< 50 vs ≥ 50 nmol/L) to provide sufficient group sizes for reliable statistical comparison. Associations with fracture status were evaluated using multivariable logistic regression models adjusted for age, sex, and BMI-z.

Logistic regression diagnostics included assessment of multicollinearity using variance inflation factors (VIFs), model fit using the likelihood ratio test and Nagelkerke’s *R*^2^, and influential observations using Cook’s distance.

To investigate whether season modified the association between serum 25OHD concentration and fracture occurrence, an additional exploratory multivariable logistic regression model including season of blood sampling (summer: May–October; winter: November–April) and a 25OHD × season interaction term was performed. The model was adjusted for age, sex, and BMI-z.

Results were expressed as odds ratios with corresponding significance levels. Receiver operating characteristic (ROC) curve analysis was conducted to evaluate the discriminatory ability of serum 25OHD for fracture occurrence.

Within the fracture cohort, comparisons between upper and lower extremity fractures were performed using Mann–Whitney *U* tests. Differences across specific fracture locations were assessed using the Kruskal–Wallis test with Dwass–Steel–Critchlow–Fligner post hoc comparisons. Associations between fracture location and age groups were evaluated using chi-square or Fisher’s exact tests.

Complete data were available for all 100 participants included in the final analytic cohort. No missing observations occurred within this cohort; therefore, no imputation was performed.

To investigate the interaction between vitamin D and calcium intake, stratified analyses were performed comparing fracture rates according to vitamin D status within low and high calcium intake groups. Statistical significance was defined as *p* < 0.05.

## Results

**Fig. 1:**
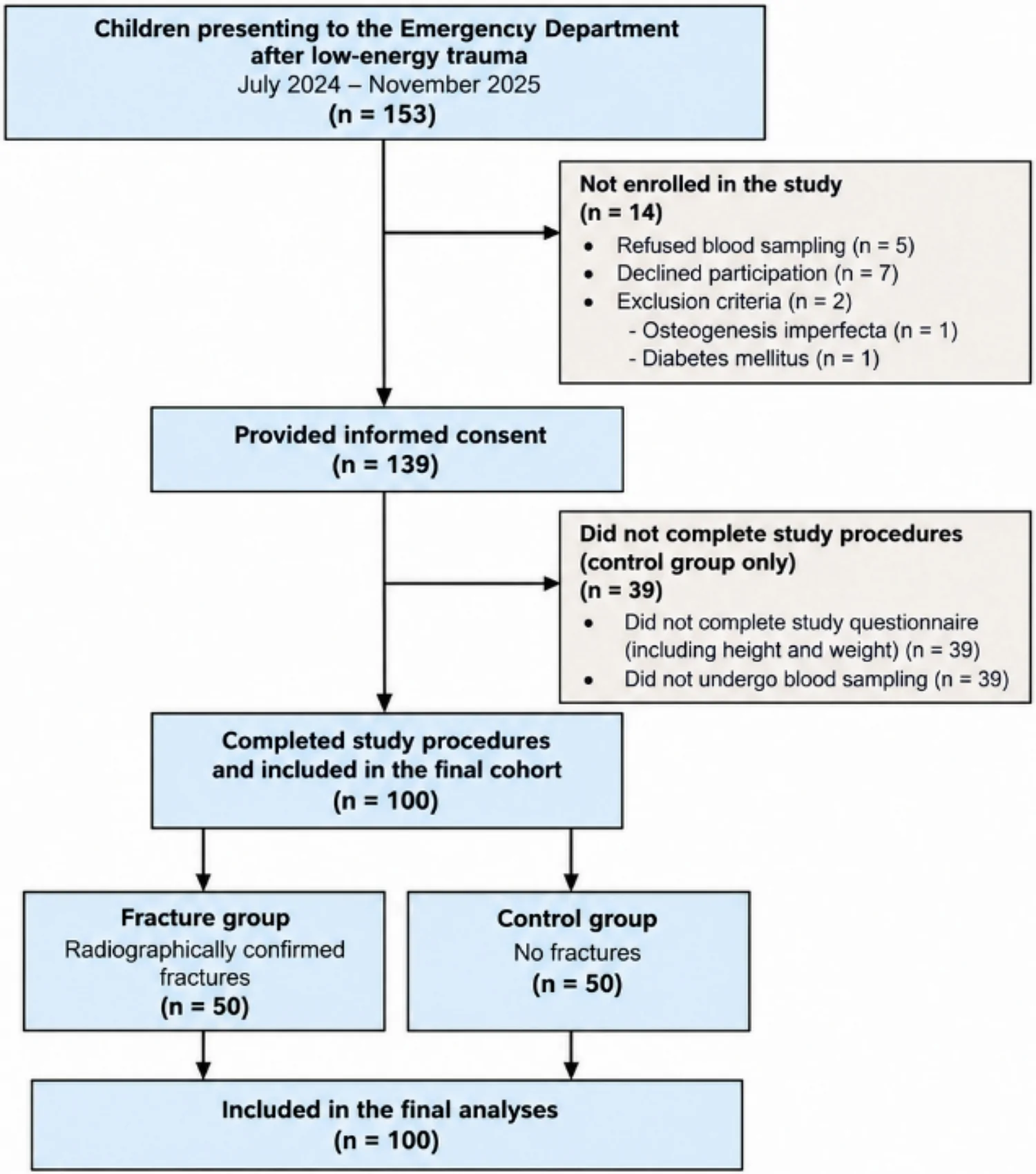
Flow diagram of participant recruitment and inclusion. Of 153 children invited to participate, 14 were not enrolled and 39 controls did not complete the study questionnaire or blood sampling. The final analytic cohort comprised 100 participants: 50 fracture cases and 50 controls

Between July 2024 and November 2025, 153 consecutive children presenting after low-energy trauma were invited to participate. Of the 139 children who initially provided informed consent, 39 controls subsequently did not complete the study procedures, as they neither completed the study questionnaire nor underwent blood sampling. They were therefore excluded before formation of the final analytic cohort. The final cohort comprised 100 participants (50 fracture cases and 50 controls). The participant recruitment process is summarized in Fig. 1.

The mean age was 10.2 (SD 3.1) years in the fracture group and 9.9 years (SD 3.3) in the control group (*p* = 0.820). Among the included fracture patients, all fractures involved the extremities; 37 affected the upper extremity (74%) and 13 the lower extremity (26%). The most common fracture locations were wrist or forearm fractures (*n* = 18) and humerus or supracondylar fractures (*n* = 13), followed by tibial fractures (*n* = 9), fractures categorized as other locations (*n* = 8), and femoral fractures (*n* = 2).

Two femoral fractures were observed: one following a fall from a small chair with entrapment of the leg, and the other during trampoline activity involving a compressive force applied to a flexed knee. Comparison of metabolic markers between fracture patients and controls revealed significantly lower 25OHD levels in fracture patients compared with controls (median 53.9 nmol/L vs 70.4 nmol/L, *p* = 0.009). No significant differences were observed for PTH (*p* = 0.314), calcium (*p* = 0.172), or phosphate levels (*p* = 0.162). In contrast, ALP levels were modestly higher in fracture patients compared with controls (median 259 vs 229 U/L, *p* = 0.038) (Table 1).

**Table 1:** Demographic, anthropometric, and biochemical characteristics of children with fractures (cases) and controls. Data are presented as mean (SD), median (min–max), or *n* (%), as appropriate. *BMI*, body mass index; *BMI z-score (BMI-z)*, age- and sex-specific body mass index z-score; *25OHD*, 25-hydroxyvitamin D; *PTH*, parathyroid hormone; *ALP*, alkaline phosphatase; *SD*, standard deviation. Height- and weight-for-age z-scores were not calculated. Race/ethnicity was not collected. *p*-values were calculated using the Mann–Whitney *U* test for non-normally distributed continuous variables, Student’s *t*-test for normally distributed continuous variables, and the chi-square test for categorical variables

Variable	Statistic	Control (*n* = 50)	Case (*n* = 50)	*p*-value
Age (years)	Mean (SD)	9.9 (3.3)	10.2 (3.1)	0.820
	Median (min–max)	10.9 (3.6–14.3)	10.6 (3.7–15.6)	
Sex, n (%)	Female	27 (54%)	16 (32%)	**0.026**
	Male	23 (46%)	34 (68%)	
Height (cm)	Mean (SD)	141.0 (20.9)	140.8 (21.2)	0.852
	Median (min–max)	148.0 (97.0–174.0)	143.5 (95.0–183.0)	
Weight (kg)	Mean (SD)	35.9 (13.4)	39.9 (15.6)	0.296
	Median (min–max)	38.8 (14.0–60.0)	38.5 (15.0–70.0)	
BMI (kg/m^2^)	Mean (SD)	17.3 (2.7)	19.4 (4.0)	**0.004**
	Median (min–max)	17.2 (12.0–26.7)	19.3 (10.4–27.5)	
BMI z-score	Mean (SD)	− 0.23 (1.00)	0.27 (1.93)	**0.006**
	Median (min–max)	− 0.16 (− 3.48 to 1.77)	0.53 (− 9.40 to 3.18)	
25OHD (nmol/L)	Mean (SD)	70.7 (21.1)	61.5 (24.0)	**0.009**
	Median (min–max)	70.4 (29.0–129.0)	53.9 (20.3–128.0)	
PTH (pg/mL)	Mean (SD)	33.9 (14.4)	37.7 (16.5)	0.314
	Median (min–max)	30.1 (11.4–91.0)	33.5 (16.4–84.0)	
Calcium (mmol/L)	Mean (SD)	2.42 (0.09)	2.40 (0.10)	0.172
	Median (min–max)	2.42 (2.22–2.75)	2.38 (2.12–2.66)	
Phosphate (mmol/L)	Mean (SD)	1.47 (0.20)	1.42 (0.19)	0.162
	Median (min–max)	1.44 (1.10–2.12)	1.44 (0.99–1.75)	
ALP (U/L)	Mean (SD)	234 (80)	271 (81)	**0.038**
	Median (min–max)	229 (76–406)	259 (107–436)	

Bold values highlight the statistical significance

**Fig. 2:**
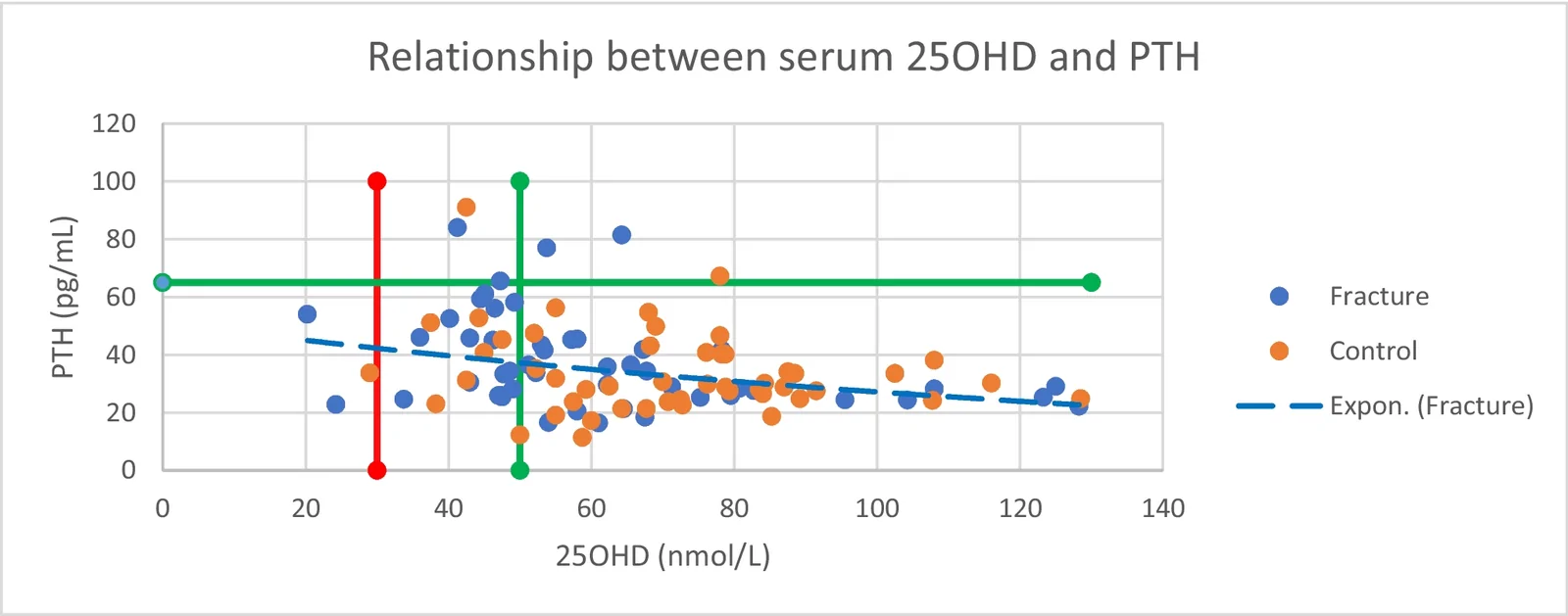
Relationship between serum 25-hydroxyvitamin D (25OHD) and parathyroid hormone (PTH). Scatter plot showing the relationship between 25OHD and PTH with an exponential trendline. Vertical lines indicate thresholds for vitamin D deficiency (< 30 nmol/L) and insufficiency (< 50 nmol/L); the horizontal line indicates the upper reference limit for PTH (65 pg/mL)

An inverse relationship between serum 25OHD and PTH was observed, consistent with physiological endocrine regulation (Fig. 2).

Vitamin D status also differed significantly between the two groups. Vitamin D insufficiency was observed in 36% of fracture patients compared with 14% of controls, whereas sufficient serum 25OHD concentrations were more frequent in controls (84%) than in fracture patients (60%) (*χ*^2^ = 7.17, *p* = 0.028). Vitamin D deficiency was uncommon in both groups.

Serum 25OHD levels were dichotomized at 50 nmol/L (< 50 vs ≥ 50 nmol/L). In multivariable analysis (Table 2), serum 25OHD concentrations below 50 nmol/L were associated with increased odds of fracture compared with sufficient levels (OR 3.27, 95% CI 1.19–8.94, *p* = 0.021). Female sex was associated with lower odds of fracture (OR 0.33, 95% CI 0.14–0.80, *p* = 0.014), while age and BMI-z were not independently associated.

**Table 2:** Multivariable logistic regression analysis of factors associated with fracture status (case vs control). Serum 25-hydroxyvitamin D (25OHD) was dichotomized at 50 nmol/L (< 50 vs ≥ 50 nmol/L). The model included sex, age, 25OHD status, and body mass index z-score (BMI-z). Male sex and 25OHD ≥ 50 nmol/L were the reference categories. *β*, regression coefficient; *SE*, standard error; *OR*, odds ratio; *CI*, confidence interval; *BMI-z*, body mass index z-score; *25OHD*, 25-hydroxyvitamin D

Predictor	Estimate (*β*)	95% CI (*β*)	SE	*Z*	*p*-value	Odds ratio (OR)	95% CI (OR)
Intercept	0.061	− 1.343 to 1.465	0.716	0.085	0.932	1.063	0.261 to 4.326
Sex (female vs male)	− 1.108	− 1.987 to − 0.229	0.449	− 2.470	0.014	0.330	0.137 to 0.795
Age (years)	0.009	− 0.123 to 0.141	0.067	0.131	0.895	1.009	0.884 to 1.151
25OHD (< 50 vs ≥ 50 nmol/L)	1.184	0.177 to 2.191	0.514	2.303	0.021	3.266	1.193 to 8.943
BMI z-score	0.233	− 0.088 to 0.555	0.164	1.423	0.155	1.263	0.916 to 1.742

Estimates represent the log odds of “Fracture status = Case” vs “Fracture status = Controls”

Regression diagnostics demonstrated no evidence of problematic multicollinearity among the predictors (all variance inflation factors ≤ 1.06). The overall logistic regression model was statistically significant (*χ*^2^ = 15.10, df = 4, *p* = 0.005), with a Nagelkerke’s *R*^2^ of 0.186. Examination of Cook’s distance identified no highly influential observations (maximum Cook’s distance = 0.659).

An exploratory multivariable logistic regression analysis including season of blood sampling and a 25OHD × season interaction term showed no evidence of effect modification by season (interaction *p* = 0.397). Likewise, season itself was not independently associated with fracture occurrence (*p* = 0.853).

**Fig. 3:**
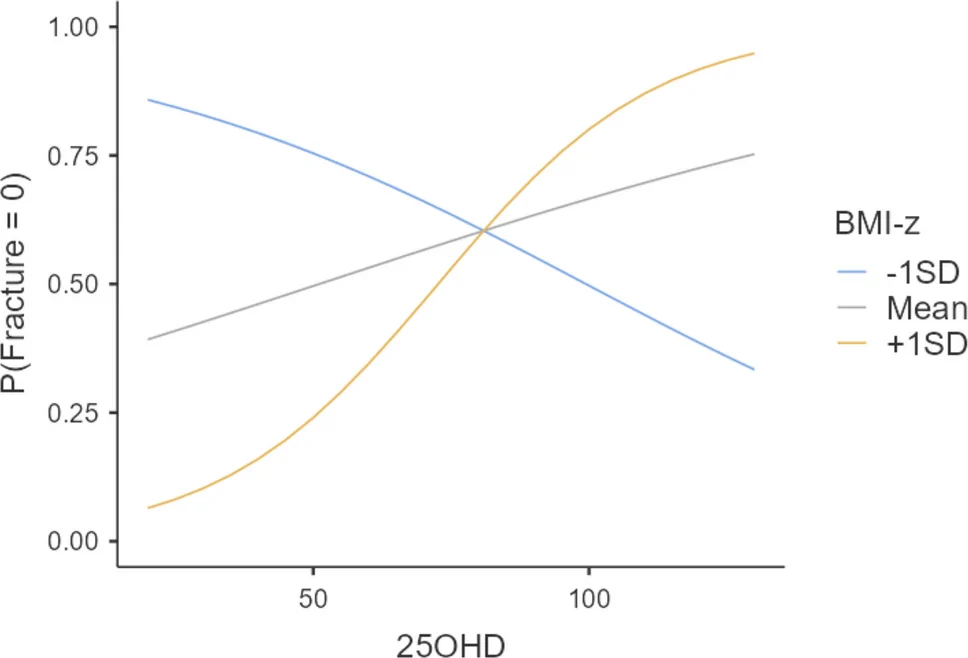
Interaction between serum 25-hydroxyvitamin D (25OHD) and body mass index z-score (BMI-z) in relation to fracture status. Predicted probabilities of no fracture [P(fracture = 0)] from the multivariable logistic regression model are shown for BMI-z values corresponding to − 1 SD, the mean (0 SD), and + 1 SD. Higher values on the *y*-axis indicate a lower probability of fracture. SD, standard deviation

When serum 25OHD was analyzed as a continuous variable, a significant interaction between 25OHD and BMI-z was observed (*β* = 0.024, *p* = 0.010), indicating that the association between serum 25OHD concentrations and fracture risk varies according to adiposity. As illustrated in Fig. 3, increasing 25OHD levels were associated with a reduced probability of fracture primarily in children with higher BMI-z, whereas this relationship was attenuated in children with lower BMI-z.

**Fig. 4:**
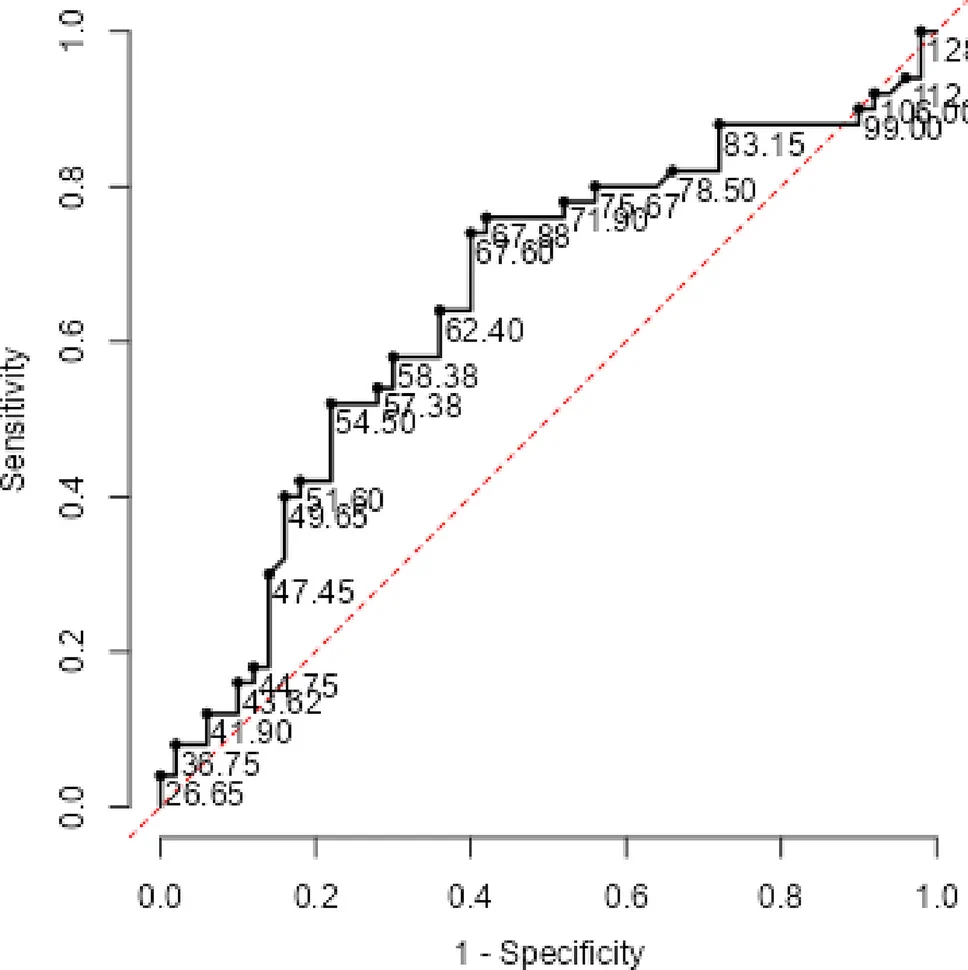
Receiver operating characteristic (ROC) curve for serum 25-hydroxyvitamin D (25OHD) concentrations in discriminating fracture cases from controls. ROC, receiver operating characteristic; 25OHD, 25-hydroxyvitamin D

ROC curve analysis demonstrated moderate discriminatory ability of 25OHD levels for fracture occurrence, with an area under the curve of 0.653 (95% CI 0.542–0.763). The optimal cutoff value identified was approximately 67.6 nmol/L, corresponding to a sensitivity of 74% and a specificity of 60% (Fig. 4).

Within the fracture cohort, no significant differences in 25OHD, PTH, calcium, or phosphate levels were observed across BMI-z tertiles. In contrast, ALP levels showed a significant difference across tertiles (Kruskal–Wallis *χ*^2^ = 7.98, *p* = 0.018). Furthermore, no significant differences in metabolic markers were observed between upper and lower extremity fractures for 25OHD (*p* = 0.407), calcium (*p* = 0.580), phosphate (*p* = 0.757), or ALP (*p* = 0.816). PTH levels were modestly higher in upper extremity fractures compared with lower extremity fractures (*p* = 0.044).

Further analysis across detailed fracture locations showed no significant differences in 25OHD (*p* = 0.411), PTH (*p* = 0.270), calcium (*p* = 0.853), phosphate levels (*p* = 0.866), or ALP (*p* = 0.653). Age differed significantly across fracture locations (Kruskal–Wallis *p* = 0.032), reflecting the known age-related distribution of pediatric fracture patterns, although pairwise comparisons revealed only limited differences between specific fracture groups.

Finally, no statistically significant association was observed between fracture location and developmental age groups (*χ*^2^ *p* = 0.307; Fisher’s exact *p* = 0.333).

Stratified analysis by calcium intake revealed a differential association between vitamin D status and fracture occurrence. In children with lower calcium intake (*n* = 49), fracture rates were significantly higher in those with low 25OHD levels compared with those with sufficient levels (78.6% vs 45.7%, *χ*^2^ = 4.36, *p* = 0.037). In contrast, among children with higher calcium intake (*n* = 51), the difference in fracture rates between vitamin D groups was smaller and did not reach statistical significance (64.3% vs 37.8%, *χ*^2^ = 2.87, *p* = 0.090). Overall, lower 25OHD levels (< 50 nmol/L) were associated with higher fracture rates (71.4% vs 41.7%, *p* = 0.008).

These findings indicate that the association between vitamin D status and fracture risk is influenced by calcium intake, with a stronger relationship observed in children with lower calcium intake.

## Discussion

This prospective case–control study identified associations between vitamin D status, adiposity, and fracture occurrence in children presenting after low-energy trauma, but the observational design does not permit causal inference. Although children with fractures exhibited lower circulating 25OHD concentrations and a higher prevalence of serum 25OHD concentrations below 50 nmol/L compared with controls, the overall discriminatory ability of 25OHD for fracture risk was modest (Fig. 4). Importantly, the association between vitamin D status and fracture risk was influenced by adiposity, indicating effect modification by BMI-z. Metabolic parameters and BMI-z were not associated with fracture location, whereas age-related differences in fracture distribution were observed.

Although femoral fractures are less commonly associated with low-energy trauma, both cases in our cohort followed clearly low-energy mechanisms, without high-energy impact: a fall from a small chair with entrapment of the leg and a trampoline-related compressive injury to a flexed knee. Both therefore fulfilled the predefined inclusion criteria for low-energy trauma.

This study observed a significant interaction between 25OHD and BMI-z, indicating that the association between 25OHD and fracture risk varied according to adiposity. The association between 25OHD and fracture risk was more pronounced in children with higher BMI-z, whereas little or no association was observed in leaner children. This finding may reflect the complex interplay between adiposity, vitamin D metabolism, and mechanical loading. In children with higher body mass, vitamin D deficiency may contribute more substantially to skeletal vulnerability, potentially due to altered bioavailability or increased mechanical stress on bone. Taken together, our results suggest that, in otherwise healthy children, fracture susceptibility is unlikely to be primarily driven by metabolic abnormalities and is more plausibly explained by biomechanical and developmental factors related to the forces exerted during falls.

These findings suggest that significant deficits in skeletal mineralisation (occult rickets and osteomalacia) are unlikely to explain fracture occurrence in this population. Previous pediatric studies have similarly reported that severe vitamin D deficiency capable of producing clinically relevant mineralisation defects is rare among otherwise healthy children living in developed countries [16–18]. Although some investigations have observed lower 25OHD concentrations in children with fractures [12, 25], systematic reviews have produced inconsistent results and have emphasized the potential influence of confounding variables such as adiposity [14]. Our data therefore support the interpretation that moderately reduced vitamin D status may be associated with fractures without necessarily representing a direct causal factor in skeletal fragility.

The expected physiological relationship between vitamin D status and PTH secretion was also evident in our dataset. As 25OHD levels declined, a compensatory increase in PTH concentrations was observed, reflecting the well-established endocrine feedback mechanism that maintains calcium balance [26, 27]. Nevertheless, PTH values remained within normal pediatric ranges for most participants, with no difference between the fracture and control group, suggesting that secondary hyperparathyroidism did not appear to be associated with fracture status in this cohort.

Recent studies [28–30] have suggested that the relationship between vitamin D status and skeletal outcomes may vary according to season because circulating 25OHD concentrations fluctuate with sunlight exposure and may therefore have different clinical implications depending on the time of blood sampling. In particular, Michaëlsson et al. [31] demonstrated that the association between low serum 25OHD concentrations and fracture risk was strongest when blood samples were obtained during periods of naturally high vitamin D levels (May–October) and among individuals with higher adiposity, suggesting that season and body composition may jointly influence the interpretation of vitamin D status. To explore this possibility, we performed an additional multivariable logistic regression including season of blood sampling and a 25OHD × season interaction term. Neither season nor the interaction term was significantly associated with fracture occurrence, indicating that the association between vitamin D status and fracture risk was not modified by season in our cohort. Collectively, these findings suggest that the modest association observed between lower serum 25OHD concentrations and fractures in otherwise healthy children cannot be explained solely by seasonal variation. It also supports the robustness of our primary findings after accounting for seasonality, although larger prospective studies are required to determine whether seasonal effects become more evident in larger pediatric populations.

An additional objective of the study was to explore whether metabolic parameters differed across fracture locations. PTH levels were modestly higher in upper extremity fractures compared with lower extremity fractures, whereas the other metabolic markers did not differ significantly by fracture region. Given the small subgroup sizes and the absence of consistent differences across detailed fracture locations, this isolated finding should be interpreted cautiously.

Consistent with this interpretation, fracture locations in our study differed across age groups. This finding aligns with established pediatric fracture epidemiology, in which supracondylar humerus fractures are more frequent in younger children, whereas distal forearm fractures become increasingly common with advancing age [1]. Changes in bone geometry, motor coordination, and patterns of physical activity during growth likely contribute to these age-dependent fracture patterns.

Bone turnover markers can provide insight into skeletal physiology during childhood [32, 33]. In the present study, the chosen mineralization markers did not differ substantially between fracture groups, suggesting no evidence of clinically relevant metabolic bone disturbance in this cohort. Since ALP levels may rise transiently following fractures as part of the normal healing response due to increased osteoblastic activity [34, 35], this likely explains the higher ALP concentrations in the fracture group.

From a clinical perspective, our findings suggest that routine measurement of serum 25OHD in children presenting with uncomplicated fractures may have limited diagnostic value. Current Endocrine Society guidelines recommend targeted testing primarily in children at known risk for rickets or other disorders of bone mineralisation rather than universal screening in all pediatric fracture cases [20].

Occasional prophylactic vitamin D supplementation was reported by some participants during the winter months; however, no child was receiving therapeutic supplementation for vitamin D deficiency or an underlying metabolic disorder.

Several limitations should be considered when interpreting these findings. First, the relatively small sample size limits statistical power, particularly for subgroup analyses according to fracture location, BMI-z, calcium intake, and vitamin D category. It also increases the uncertainty of interaction estimates and may have prevented the detection of smaller associations. Second, although participants were recruited prospectively, the case–control design does not establish temporality or causality. Serum biochemical parameters were measured within 7 days after injury rather than before the traumatic event. Consequently, post-injury physiological responses, particularly changes in alkaline phosphatase related to early fracture healing, may have influenced some measurements, preventing them from being interpreted as unequivocal pre-fracture values. Third, residual confounding cannot be excluded. Although the analyses considered age, sex, BMI-z, calcium intake, and season of blood sampling, other potentially relevant factors—including pubertal stage, habitual physical activity, sunlight exposure, socioeconomic factors, and bone mineral density—were not comprehensively measured. The distribution of blood sampling across seasons did not differ significantly between fracture cases and controls; nevertheless, residual confounding related to seasonal sunlight exposure and outdoor activity remains possible. Fourth, dietary calcium intake was estimated using a parent-completed food frequency questionnaire and therefore may be subject to recall bias and reporting inaccuracies.

An additional limitation is that 39 controls who initially provided informed consent did not complete the study questionnaire or undergo blood sampling and were therefore not included in the final analytic cohort. As individual-level data for these non-completers were no longer available for comparison with included controls, potential selection bias related to differential study completion cannot be excluded.

These limitations mean that the observed associations should not be interpreted as evidence that low 25OHD concentrations or adiposity directly caused the fractures.

## Conclusions

In this case–control study, children presenting with fractures after low-energy trauma exhibited lower circulating 25OHD levels and a higher prevalence of vitamin D insufficiency compared with controls. However, biochemical signs of impaired mineralisation were largely within normal ranges, with the exception of ALP, and no evidence of a clinically relevant mineralisation defect was identified.

While vitamin D insufficiency was associated with increased odds of fracture, its effect was not uniform across the study population. When analyzed as a continuous variable, the association between 25OHD and fracture risk was modified by BMI-z, suggesting that vitamin D may play a more relevant role in children with higher adiposity.

Overall, our findings suggest that fracture susceptibility in otherwise healthy children is predominantly influenced by biomechanical and developmental factors, while vitamin D status appears to act as a modest modifier, particularly in children with greater adiposity. Routine laboratory screening for metabolic abnormalities, including 25OHD measurement, therefore has limited value in uncomplicated pediatric fractures, although targeted assessment may be considered in selected subgroups.

Future, prospective studies integrating biochemical markers, imaging or bone biopsy-based assessments of bone strength, and detailed evaluation of injury mechanisms may further clarify the determinants of fracture susceptibility in pediatric populations.

## Data Availability

The data that support the findings of this study are available from the corresponding author upon reasonable request.
